# P11 Re-evaluating a historical Indigenous Mesoamerican wound remedy: antibacterial and anti-biofilm activity of agave syrup

**DOI:** 10.1093/jacamr/dlag102.017

**Published:** 2026-06-26

**Authors:** Ross Pallett, Kathleen Pritchard, Kayleigh Wilkins, Raphael Galleh, Olusoji Adebisi, Lucy Harman, Talvin Momi, Harvey Wilkes, Caroline Dodds Pennock

**Affiliations:** Department of Biosciences, School of Medicine, Pharmacy and Biosciences, Aston University, UK; Department of Biosciences, School of Medicine, Pharmacy and Biosciences, Aston University, UK; Department of Biosciences, School of Medicine, Pharmacy and Biosciences, Aston University, UK; Institute of Science and Environmental Science, University of Cumbria, UK; Department of Biosciences, School of Medicine, Pharmacy and Biosciences, Aston University, UK; Department of Biosciences, School of Medicine, Pharmacy and Biosciences, Aston University, UK; Department of Biosciences, School of Medicine, Pharmacy and Biosciences, Aston University, UK; Department of Biosciences, School of Medicine, Pharmacy and Biosciences, Aston University, UK; Department of History, University of Sheffield, UK

## Abstract

**Background:**

Antibiotic-resistant infections emphasize the urgent need to identify and test novel antimicrobial agents. Numerous areas are currently under investigation, including antimicrobial peptides, bacteriophage therapy, and plant-derived compounds. The re-evaluation of historical remedies for their antimicrobial activity is another promising avenue of research. The *Agave* plant held significant social, religious, and medicinal value for Indigenous Mesoamerican communities, including the Mexica (Aztecs) as documented in the *Florentine Codex*, a sixteenth-century text authored by Spanish Franciscan friar Bernardino de Sahagún.

**Objectives:**

To honour the historical use of agave syrup in the treatment of wounds and infections by evaluating the antimicrobial activity of five commercially available syrups, primarily derived from *Agave tequilana*.

**Methods:**

Commercial syrups were selected as a readily accessible and standardized material for laboratory testing. MICs were determined using the microbroth dilution method, whilst anti-biofilm activity was assessed using crystal violet staining.

**Results:**

Our findings demonstrate that agave syrups significantly inhibit the planktonic growth of six clinically relevant organisms known to cause wound infections. These included the Gram positive bacteria *Staphylococcus aureus, Staphylococcus epidermidis and Streptococcus pyogenes,* as well as the Gram negative bacteria *Pseudomonas aeruginosa, Escherichia coli* and *Acinetobacter baumannii.* The traditional practice of mixing the agave syrup with salt as detailed in the codex enhanced the syrups’ antibacterial efficacy against *E. coli*. Moreover, the Agave syrups significantly inhibited biofilm formation across all bacterial species tested, although their ability to disrupt pre-formed biofilms appears to be species specific.

**Conclusions:**

Investigations into the antimicrobial mechanisms suggests that the syrups activity arises from a combination of factors, including acidic pH, the presence of the dicarbonyl compound methylglyoxal, and hydrogen peroxide, all of which are known to contribute to the antimicrobial activity of honey. Overall, these findings support the growing reappraisal of historical infection remedies and highlight the value of ethnopharmacology and Indigenous knowledge in identifying novel antimicrobial agents.

